# Research on prediction model of converter temperature and carbon content based on spectral feature extraction

**DOI:** 10.1038/s41598-023-41751-9

**Published:** 2023-09-02

**Authors:** Bo Zhao, Jinxuan Zhao, Wei Wu, Fei Zhang, Tonglu Yao

**Affiliations:** 1 Metallurgical Technology Institute, Central Iron and Steel Research Institute Co. Ltd., Beijing, 100081 People’s Republic of China; 2 HBIS Group Co. Ltd., Shijiazhuang, 050023 People’s Republic of China

**Keywords:** Optical spectroscopy, Scientific data

## Abstract

The flame of converter mouth can well reflect the change of temperature and composition of molten steel in the furnace. The flame characteristics of converter mouth collected by device can well predict the smelting process of converter. Based on the flame spectrum data set of converter mouth, this paper uses the BEADS algorithm and rough set attribute reduction algorithm optimized by genetic algorithm to extract the features of 2048-dimensional wavelength data. Through the model, eight indexes that contribute greatly to temperature and carbon content are selected, which are *f*-507, *f*-520, *f*-839, *f*-1073, *f*-1371, *f*-1528, *f*-1727 and *f*-1826. The MIC coefficients of the eight indicators with temperature and carbon content are calculated, and the MIC coefficients of the variables is small, and the selected indicators are representative. There was a significant correlation between temperature and C content. In BP neural network of temperature prediction model, it is found that the prediction accuracy of the training set is 0.99, the prediction accuracy of the test set is 0.99, the prediction accuracy of the verification set is 0.99, and the prediction accuracy of the whole set is 0.99. Through statistics, it is found that the hit rate of the temperature model in the range of ± 5 K is 88.7%, and the hit rate in the range of ± 10 K is 98.4%. and the *RMSE* parameter analysis shows that the average prediction error is 3.85 K. In BP neural network of carbon content prediction model, it is found that the prediction accuracy of the training set is 0.99, the prediction accuracy of the test set is 0.99, the prediction accuracy of the verification set is 0.98, and the prediction accuracy of the whole set is 0.99. Through statistics, it is found that the hit rate of the carbon contents model in the range of ± 0.05% is 94.0%, and the hit rate in the range of ± 0.10% is 98.3%, and the *RMSE* parameter analysis shows that the average prediction error is 0.021%. Finally, the universality of the model is verified by MIV algorithm.

## Introduction

At present, the converter steelmaking process is the main means to obtain steel. The more uniform and stable the converter composition and temperature of the steel, the lower the requirements for subsequent treatment, and the smelting cost will be reduced accordingly. Meanwhile, the appropriate tapping steel with high carbon guidance for the realization of the steel industry ' carbon neutral ' is very meaningful^1–5^. This requires accurate model for the prediction of converter end point. Generally, carbon content and temperature are thought as the criteria for judging whether the end point of smelting is reached. There are mainly static models and dynamic models for the prediction of the carbon content and temperature. The static model is to calculate the weight of molten iron, the number of auxiliary materials, the amount of oxygen blowing and other indicators^6–8^. Through the calculation of material balance and heat balance, the theoretical C content and temperature of molten steel are calculated. The dynamic model is to use the information of the sub-gun sampling intermittently at the moment when the converter smelting is close to the end point and correct the oxygen supply and cooling dose of the molten pool calculated by the static model to determine whether the end point is reached^7,9^. Because there are many kinds of steel that can be smelted by converter, the process control is not the same.

Scholars have made a lot of achievements in the research of converter static model and dynamic model. Martin^10^ studied the application accuracy of the static model. The model was more accurate in predicting the equilibrium P content, but the accuracy was not enough in predicting the C content and temperature. The average error of temperature prediction was 19.5 °C, and the average error of C content prediction was 0.0128%. Wang^11^ used the static input model combined with the hybrid model of ANN algorithm to predict the end point temperature, carbon, and phosphorus by taking the chemical composition of molten iron, process parameters and auxiliary material addition as input. By collecting the data of 28,000 furnaces of Tata Company in India for training, the fluctuation range of temperature prediction accuracy is about 53 °C, and the fluctuation range of C prediction accuracy is about 0.013%. Shi^12^ compared the furnace gas analysis control method with the manual experience steelmaking control, it is found that the endpoint carbon temperature hit rate is 94.27% and 90.26% respectively, and the double hit rate is 86.54%. The static model only considers the thermodynamic aspect, and often ignores the situation that the kinetic conditions cause insufficient reaction, and the predicted value is often quite different from the actual.

At the smelting site, experienced operators rely on the colour of the furnace mouth flame to determine the temperature and carbon content, which provides a new idea for converter endpoint prediction. The colour of the flame can present the intersection temperature, and the flame colour is related to the CO content, which makes the end point prediction become an image recognition problem. The shot was used to view the flame colour, and the optical information emitted from the flame is projected onto the photoelectric detector by pinhole imaging theory, and the light intensity data of the flame are intermittently recorded by the photoelectric detector according to the discrete frequency. Shao^13^ used SVC and SVR algorithms to establish a model of spectral characteristics and end-point C content for the spectral data collected by the furnace flame, and the model accuracy was high. By extracting the spectral characteristics of the furnace flame, Zhou^14^ selected the more representative spectrum for analysis and used it as the input of the support vector machine. Combined with the decarburization theory and the measured carbon value, the reconstructed decarburization function curve was fitted as the output of the support vector machine. The detection accuracy of the end-point carbon content was 90.2%. The existing research of scholars can fully explain the practicability of spectral prediction of converter temperature and carbon content, but the accuracy of current spectral research is still not very high^15–19^. The significance of the prediction model is to improve the accuracy, and the accuracy of about 90% is not much reference compared to the operator, so it is necessary to improve the prediction accuracy.

Aiming at the problem of inaccurate prediction of converter temperature and carbon content and the application of big data technology in metallurgical industry, based on the spectral big data dataset, this paper uses the attribute reduction algorithm optimized by genetic algorithm to extract the spectral dimension features, selects the wavelengths that have a greater impact on temperature and carbon content, and then uses the 3σ principle to eliminate the abnormal values of the spectrum to ensure the accuracy of the data. Finally, the BP neural network algorithm is used to establish a dynamic prediction model for temperature and carbon content, which provides a new idea for converter end point control.

## Mathematical model

### Collection of spectral data

**Figure 1:**
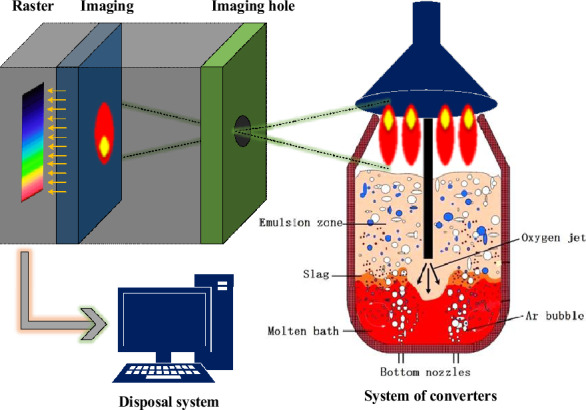
Optical data generation process at a certain time.

The spectral data used in this paper are from the steel plant. Due to the safety of operation and the limitation of experimental cost, the flame data of one furnace is collected. To make the carbon content data changes more widely distributed, the selected steel grade is high carbon steel (82B). In the actual spectral data acquisition process, select the USB4000 spectrometer to collect the spectral information of furnace flame. The data content is the reflectivity under the uniform distribution of the corresponding wavelength of 340.54 ~ 923.68 nm (the spectrometer can collect > 700 nm near-infrared wavelengths that are not visible to the human eye, allowing flame characteristics to be retained to a greater extent), and the step size of wavelength segmentation is 0.285 nm, so the dimension of one-time acquisition data is 2048. The data are collected every 0.5 s. In the early stage, 201.5 s data was taken, 143.5 s data was taken in the middle stage, and 142.5 s data was taken in the later stage. Optical information data generation process at a certain time as shown as Fig. 1.

**Figure 2:**
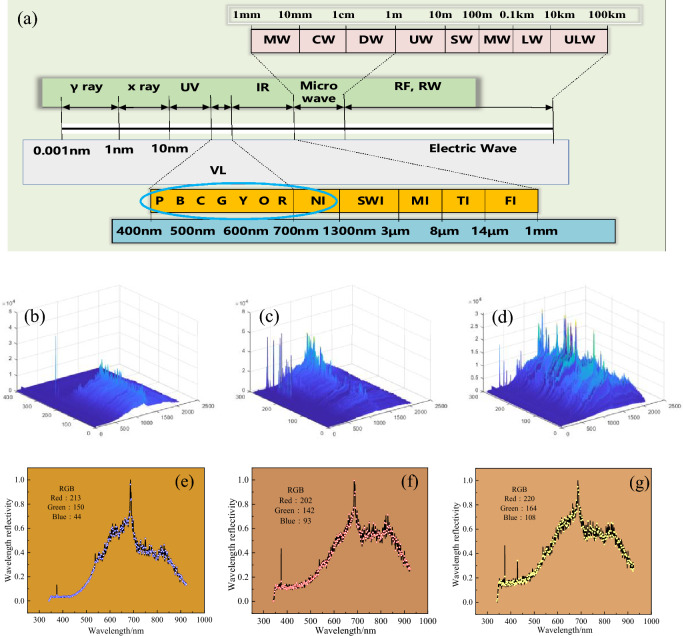
Spectral light intensity statistics of converter in different smelting periods. (**a**) Electromagnetic spectrum diagram (**b**) Spectral light intensity at early stage of converter smelting; (**c**) Spectral light intensity at middle stage of converter smelting; (**d**) Spectral light intensity at late stage of converter smelting; (**e**) Initial spectral typical color (RGB); (**f**) Medium spectral typical color (RGB); (**g**) Late spectral typical color (RGB).

A total of 403 sets of data in the early stage, 287 sets of data in the middle stage and 285 sets of data in the later stage were collected by the device, Normalize the light intensity, and convert the wavelength reflectance to RGB tristimulus colour by algorithm, the data collected is shown in Fig. 2.

The data of flame spectrum acquisition in the early and middle stages are shown in Fig. 2b–d. To describe more specifically, a set of typical spectra from the songs of the middle and late periods are explained, as shown in Fig. 2e–g. After the spectrometer converts the flame color into a spectrum, two sets of data are formed. One is the spectral wavelength, that is, the abscissa of the graph, the unit is nm, and the other is the wavelength reflectance, that is, the ordinate of the graph. Through comparison, it is found that the spectral light intensity of the three periods is very different and specific. The background color of Fig. 2e–g is flame color, which is represented by RGB values. The early flame color is orange, the flame is brighter; the color of the middle flame is orange, and the flame is dark; the later flame color is whiter than before, and the flame restores its bright color. Through the color intuitive performance of the flame changes in each period.

### Removal of spectral curve baseline

It can be seen from Fig. 2 that the reflectance of different wavelengths of the spectrum is very different, which makes the change range of each wavelength not easy to detect. Therefore, the baseline of the spectrum needs to be removed to provide the possibility for the feature extraction of the wavelength. The algorithm is implemented by MATLAB third-party toolbox (BEADS, Baseline estimation and sparse noise reduction)^20^. The main functions of the algorithm are baseline correction, noise reduction and instrument zero drift correction. There is a limitation that the signal to be processed must be a sparse signal in which most of the peaks are positive. The BEADS algorithm transforms the spectral baseline fitting problem into a convex optimization problem, and uses an asymmetric penalty function and a robust, computationally efficient iterative algorithm to ensure convergence to a unique optimal solution. The solution function is shown in Eqs. (1)–(3).

1 $$[\hat{x},\hat{f},COST] = {\text{beads}}(y,d,f_{c} ,r,\lambda_{0} ,\lambda_{1} ,\lambda_{2} )$$

2 $$\hat{x} = \arg \mathop {\min }\limits_{x} \left\{ F(x) = \frac{1}{2}||H(y - x)||_{2}^{2} + \lambda_{0} \sum\limits_{n = 0}^{N - 1} {\theta}_{\varepsilon } (x_{n} ;r) + \sum\limits_{i = 1}^{M} {\lambda_{i} \sum\limits_{n = 0}^{{N_{i} - 1}} {\phi ([D_{i} x]_{n} )} } \right \}$$

3 $$\hat{f} = L(y - \hat{x})$$

where, *y* is the observed value, *x* is the peak value of the spectral absorptivity, *f*_*c*_ is the cutoff frequency, *r* is the asymmetric parameter, *λ*_*i*_ is the regularization parameter, *F* is the cost function, *H* is the high-pass filter, *θ* is the asymmetric penalty function, *φ* is the symmetric penalty function, *D*_*i*_ is the *i*-order difference operator, *L* is the low-pass filter, $\hat{x}$ is the spectral absorptivity value for removing the baseline, $\hat{f}$ is the baseline.

The parameter settings of the BEADS model are shown in Table 1.

**Table 1:** Parameter settings of the BEADS model.

Parameters	Values	Parameters	Values
*f*_*c*_	0.08	*λ*_0_	0.1
*d*	1	*λ*_1_	1
*r*	6	*λ*_2_	0.8

### Spectral feature screening

If considering 2048 light intensity data generated at a time as the input and the flame temperature and the C element content as the output, constructing a mathematical model is bound to be complex, so that it can not achieve the forecast results. In order to reduce the number of input data, try to find one or more characteristic values of light intensity data, which will be used as input to reduce the complexity of the model calculation, and enhance the applicability of the model.

Index dimension reduction methods commonly used will be mainly factor analysis, principal component analysis, etc., but these methods belong to the mapping dimension reduction, that is, to create new indicators to cover the original indicators, but these indicators do not exist obvious physical meaning, that is, there is no obvious effect on the optimization. The dimensionality reduction of spectral data in this paper refers to the selection of indicators with large changes and strong correlation with predictive variables, and these indicators have obvious physical significance. Therefore, the rough set attribute reduction is a very suitable choice. Rough set theory can remove redundant information in data under the premise of maintaining the original classification ability. In the same time, Genetic algorithm can be used to adaptive global optimization which simulates the genetic and evolutionary process of organisms in the natural environment. Using the attribute reduction method combining rough set theory and genetic algorithm to remove redundant rules in the decision table can effectively reduce the index dimension and obtain accurate feature representation.

**Figure 3:**
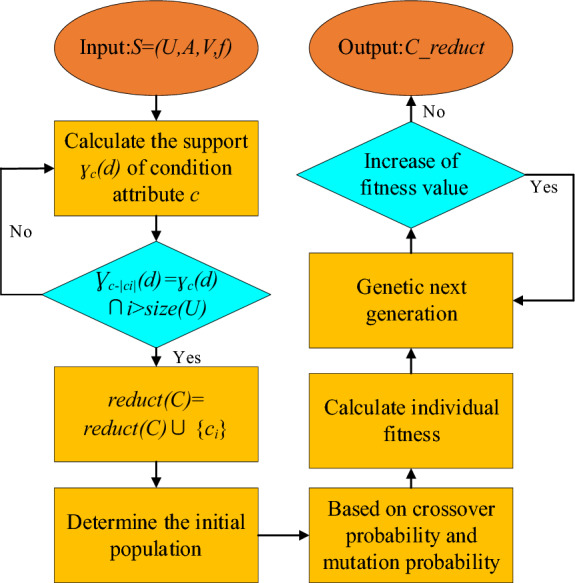
Steps of rough set algorithm for genetic algorithm optimization.

The dependence of decision attribute *A* on condition attribute *V* is shown in Eq. (4), and the optimization function is shown in Eq. (5). The specific steps of GA optimized rough set algorithm are shown in Fig. 3.

4 $$\gamma_{V} (A) = \frac{{|POS_{V} (A)|}}{|U|}$$

5 $$F(r) = @TargetOptFun\left( {U,A,V,f} \right) = \frac{{n - l_{r} }}{l} + \gamma_{s} (d)$$

where *U* is the domain of discourse, *A* is the conditional attribute, *V* is the decision attribute, *f* is the fitness function, *γ*_*s*_ is the attribute dependence, and *C* is the conditional attribute after *y* reduction. The parameter settings of the GA optimized rough set model are shown in Table 2.

**Table 2:** Parameter settings of the GA optimized rough set model.

Parameters	Values
*p*_*c*_	0.7
*P*_*m*_	0.01
*Max_Gen*	150

### BP neural network algorithm basis

**Figure 4:**
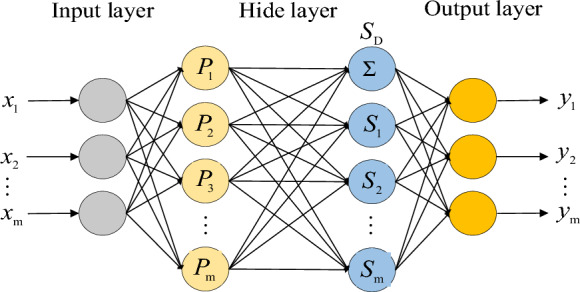
Neural network structure.

Through the iterative process of the algorithm, the error signal meets the engineering setting threshold requirements. The specific network structure is shown in Fig. 4. Where n represents the number of neurons in the input layer, *p* and *q* represent the number of neurons in the hidden layer, *m* represents the number of neurons in the output layer. *x*_*i*_ denotes the input of the *i*-th neuron in the input layer, the output of the *j*-th neuron in the hidden layer, and the output of the *k*-th neuron in the output layer. *w*_*ij*_ and *w*_*jk*_ represent the weights of the *i*-th neuron in the input layer to the *j*-th neuron in the hidden layer, and the weights of the j th neuron in the hidden layer to the *k*-th neuron in the output layer, respectively. *θ*_*j*_ and *θ*_*k*_ represent the threshold of the hidden layer neuron and the output layer neuron respectively. Represents the activation function of the hidden layer and represents the activation function of the output layer.

The establishment process of BP neural network model is mainly divided into the following steps.

#### Step 1: forward propagation process of signal

The output of the *j*-th neuron in the hidden layer is:

6 $$y_{j} = f \left(\sum\limits_{i = 1}^{n} {w_{ij} x_{i} + \theta_{j} } \right ) = f(net_{j} )$$

where, $net_{j}$ denotes the input of the jth neuron in the hidden layer.

The output function of the *k*-th neuron in the output layer is:

7 $$z_{k} = g \left(\sum\limits_{i = 1}^{n} {w_{jk} y_{j} + \theta_{k} } \right) = f(net_{k} )$$

where, $net_{k}$ denotes the input of the kth neuron in the hidden layer.

If $o_{k}$ is used to represent the expected error of the *k*-th neuron in the output layer, the error between the actual output and the expected output of the *k*-th neuron is:

8 $$e_{k} = o_{k} - z_{k}$$

The total error of network output is:

9 $$E = \frac{1}{2} \left(\sum\limits_{I = 1}^{q} {o_{k} - z_{k} } \right) = \frac{1}{2}\sum\limits_{k = 1}^{q} {e_{k}^{2} }$$

If the total error *E* satisfies the termination condition of the network, the training is completed; otherwise, the weights and thresholds of the network are adjusted repeatedly using the back propagation of the error signal until *E* satisfies the termination training objective.

#### Step 2: the back propagation process of error signal

In the back propagation process of the error signal, the gradient descent method is used to adjust the weights and thresholds of each layer of neurons, so that the output error of the adjusted neural network is closer to the expected error. If ${\mathrm{d}}_{j}$ is the output error of the *j*-th neuron in the hidden layer, the expression is:

10 $$d_{j} = \sum\limits_{k = 1}^{q} {e_{k} \cdot \theta_{k} \cdot f \left(\sum\limits_{i = 1}^{n} {w_{ij} y_{i} + \theta_{j} } \right )}$$

The threshold of the output layer is adjusted to:

11 $$\theta_{k} (k + 1) = \theta_{k} (k) + \eta \cdot e_{k}$$

The threshold adjustment of the hidden layer is:

12 $$\theta_{j} (k + 1) = \theta_{j} (k) + \eta \cdot d_{j}$$

The connection weights between the output layer and the hidden layer are adjusted to:

13 $$w_{jk} (k + 1) = w_{jk} (k) + \eta \cdot e_{k} \cdot y_{i}$$

**Figure 5:**
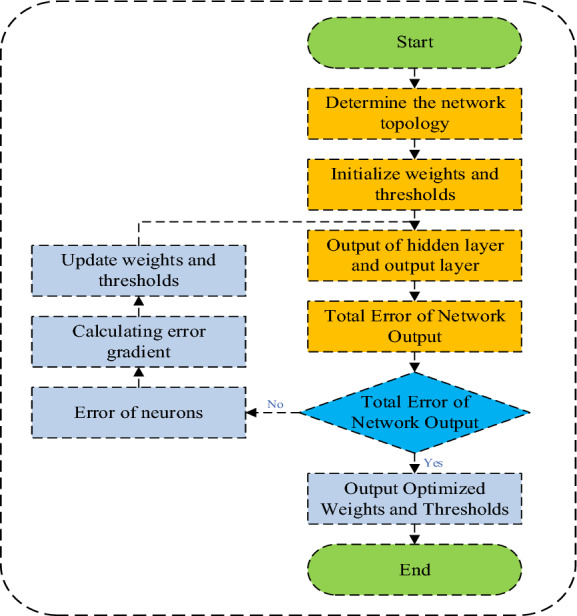
BP neural network algorithm flow chart.

The connection weights between the input layer and the hidden layer are adjusted to:

14 $$w_{ij} (k + 1) = w_{ij} (k) + \eta \cdot d_{j} \cdot x_{i}$$

where, *η* is the learning rate of the neural network. According to the above analysis, the specific process of BP neural network learning algorithm can be represented by Fig. 5.

Finally, the results of the neural network show the following nonlinear regression equation. The meaning of the parameters has been explained above:

15 $$\begin{aligned} simy = & w_{11}^{23} \times \tan sig(w_{11}^{12} \times x_{1} + w_{21}^{12} \times x_{2} + \ldots + w_{m1}^{12} \times x_{m} + b_{1}^{(2)} ) \\ & + \;w_{21}^{23} \times \tan sig(w_{12}^{12} \times x_{1} + w_{22}^{12} \times x_{2} + \ldots + w_{m2}^{12} \times x_{m} + b_{2}^{(2)} ) \\ & + \;w_{31}^{23} \times \tan sig(w_{13}^{12} \times x_{1} + w_{23}^{12} \times x_{2} + \ldots + w_{m3}^{12} \times x_{m} + b_{3}^{(2)} ) \\ & + \;b_{1}^{(3)} \\ \end{aligned}$$

#### Step 3: models checkout

The test method used in this paper is to compare the predicted value with the actual value, which involves an important parameter is *RMSE*. *RMSE* is defined as the mathematical expectation of the square of the difference between the estimated value and the true value of the parameter, which is used to measure the deviation of the data. The specific calculation method is as follows:

16 $${\text{RMSE}} = \left[ {\frac{{1}}{{\text{N}}}\sum\limits_{i = 1}^{N} {(y_{i} - \overset{\lower0.5em\hbox{$\smash{\scriptscriptstyle\frown}$}}{y}_{i} )^{2} } } \right]^{1/2}$$

For the eliminated data, eight independent variables obtained by the above rough set theory are used as input variables of BP neural network. The input nodes of the neural network are set to *m*, and the output nodes are set to *n* (Temperature/Carbon Contents). The empirical formula for calculating the number of hidden layer nodes in the network is: j = (m + n)^0.5^ + α (m is the number of neurons in the input layer, n is the number of neurons in the output layer), so after calculation, the hidden layer nodes are set to 17.

For the setting of the learning rate and the number of hidden layers of BP neural network, this paper selects the grid search method to optimize, and uses the enumeration method to optimize the iterative process. The learning rate is set to 0.01,0.1,1 and 10, and the number of hidden layers is 1, 2, 3, 4 and 5 respectively. Through cyclic traversal, the parameters of each group are calculated, and the best performance parameter is the final result. In this paper, RMSE is used as the optimization index, and the calculation results are shown in Table 3.

**Table 3:** The accuracy of the model with different learning rates and the number of hidden layer nodes.

Model	Learning rate	5	7	9	11	13
Temperature model	0.01	3.5075	3.8322	3.1748	3.9582	3.2699
Carbon content model		2.6372	2.5884	2.9432	2.9965	3.3001
Temperature model	0.1	3.3523	3.4694	3.9672	3.3906	4.6371
Carbon content model		2.6651	2.6335	2.6816	2.9362	2.7101
Temperature model	0.5	3.1125	3.4981	3.5089	3.7421	4.3108
Carbon content model		2.7069	2.7209	2.6790	3.1528	3.0684
Temperature model	0.75	3.4271	3.5591	3.6988	4.2017	3.4565
Carbon content model		2.8686	2.4163	2.4612	2.8385	2.9074
Temperature model	0.875	3.3052	3.0597	3.2753	3.6633	4.4145
Carbon content model		2.6664	2.7993	2.6601	2.6182	2.9970
Temperature model	1	3.1154	3.3338	3.0376	3.8678	3.5045
Carbon content model		2.1175	2.6101	2.4359	2.6747	2.7283
Temperature model	10	3.1842	3.6801	3.8684	3.4829	4.1153
Carbon content model		2.7873	2.7208	2.7496	2.4447	2.6626

Through the enumeration results, it can be found that when the learning rate is set to 1 and the number of hidden layer nodes is set to 5, the *RMSE* of the model is lower. Therefore, this paper establishes the model with this parameter setting.

## Results and discussion

### Baseline removal results

**Figure 6:**
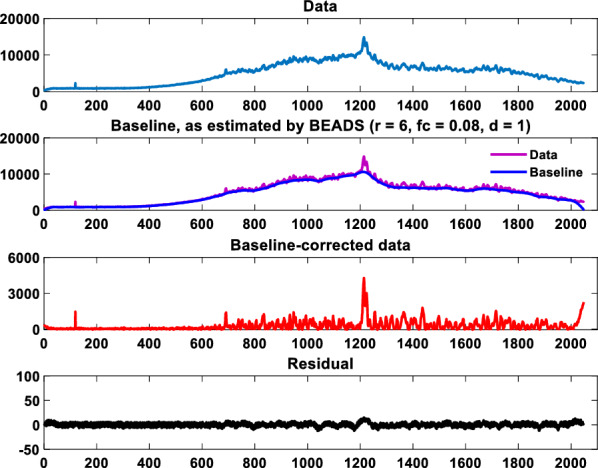
BEADS algorithm removes spectral baseline effect map (the first group datas).

One set of spectral data was processed, and the processing effect is shown in Fig. 6. It can be seen that the BEADS algorithm fits the spectral baseline very well and preserves the broad-spectrum characteristic peaks completely. At the same time, the data of the middle and late stages are analyzed, and the analysis effect is the same as the data of the initial stage of smelting. Therefore, it is considered that the spectral information is well preserved.

### Index attribute reduction results

**Figure 7:**
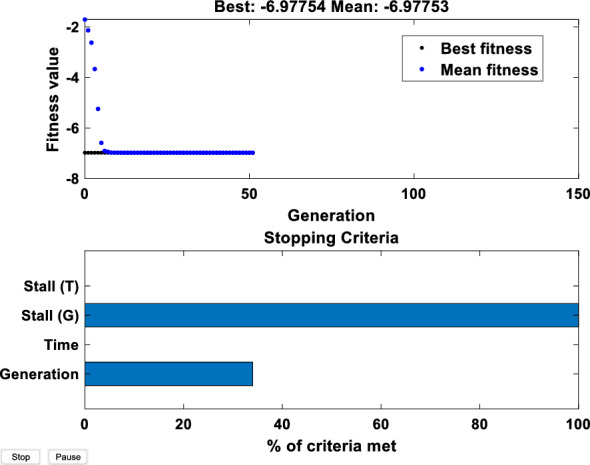
Iterative process of genetic algorithm.

Through attribute reduction of 978 sets of data, converges at the 50th iteration, and the iterative process is shown in Fig. 7.

It can be seen from the iterative results that the fitness function converges after the 7-th iteration, and there is no obvious change after that, so the algorithm results are considered to be more reliable. After attribute reduction, the spectral wavelength with strong importance can be obtained. The results are shown in Table 4.

**Table 4:** Variables after attribute reduction.

Variable	*X*_*1*_	*X*_*2*_	*X*_*3*_	*X*_*4*_	*X*_*5*_	*X*_*6*_	*X*_*7*_	*X*_*8*_
Wavelength dimension	*f*-507	*f*-520	*f*-839	*f*-1073	*f*-1371	*f*-1528	*f*-1727	*f*-1826
Corresponding wavelength (nm)	485	488.7	579.6	646.3	731.3	776	832.7	861
Relevance grade	− 6.978							

**Figure 8:**
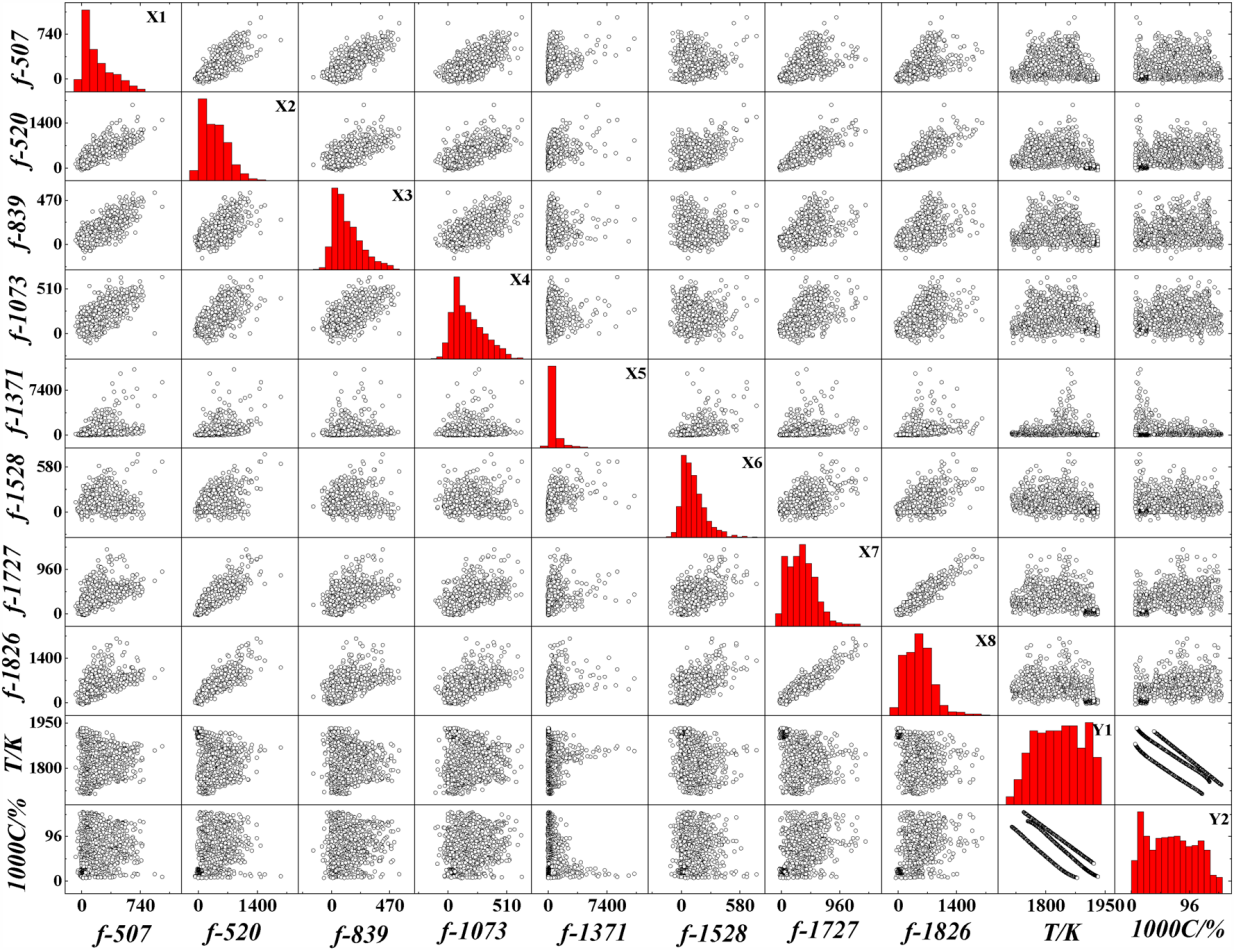
Data range and statistical histogram of independent variables and dependent variables.

To avoid the low accuracy of the model caused by the fluctuation of single variable data, the average value of the upper and lower 10 variables are used as the index data. The spectral acquisition data of one smelting cycle are collected, and the variation range and statistical histogram of each variable are shown in Fig. 8. It can be seen from the analysis that the distribution width and breadth of each index are not the same, indicating the specificity of each index. When conducting machine learning training, it is often hoped that the closer the data is to the normal distribution, the better, so that the training effect will be significantly improved. Therefore, this paper uses the Jarque–Bera method to test the normal distribution of the data. The test results are shown in Table 5.

**Table 5:** Normal distribution test results.

Variables	Skewness	Kurtosis	Variables	Skewness	Kurtosis
*f*-507	0	0	*f*-1528	0	0.0036
*f*-520	0	0	*f*-1727	0	0.0132
*f*-839	0	0	*f*-1826	0	0
*f*-1073	0	0	Temperature	0.3557	0
*f*-1371	0	0	C Contents	0.0558	0

Through the normal distribution test, it was found that the index skewness of spectral data screening was 0, and the kurtosis < 0.05, so it was considered to be normal distribution. The skewness of the temperature data is 0.3557 < 1, and the kurtosis is 0, which is considered to be approximately normal distribution. The skewness of the carbon content data is 0.0558, and the kurtosis is 0, so the data is considered to obey the normal distribution.

The correlation analysis of the selected indicators shows that the relationship between spectral wavelength and temperature and carbon content is nonlinear, so the conventional Pearson coefficient is not suitable. In this paper, the MIC model is selected to calculate the correlation between the indicators, and the calculation formula is shown in Eq. (17).

17 $$MIC(x,y) = \mathop {\max }\limits_{|x||y| < B} \frac{I(x,y)}{{\log_{2} (\min (|x|,|y|))}}$$

where, MIC is the MIC value between the indicators, x and y are two random variables of correlation analysis, and I is the mutual information between the variables. The calculation formula is shown in Eq. (18).

18 $$I(x,y) = \sum\limits_{x,y} {p(x,y)\log_{2} \frac{p(x,y)}{{p(x)p(y)}}}$$

**Figure 9:**
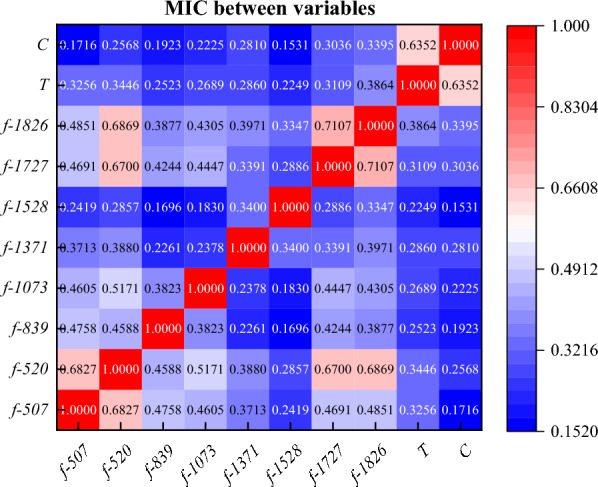
MIC coefficient between indicators.

The calculation results are shown in Fig. 9. It can be seen from Fig. 9 that the correlation between some indicators is very strong. Combined with Fig. 7, this part of the indicators show a unified upward trend, which is the main reason for the high degree of collinearity. It can be seen from the MIC coefficients of the index after attribute reduction and temperature and C content that there is a certain correlation between them, indicating that it is feasible to predict the end point temperature and C content of the converter by spectrum.

### Data filtering and elimination

First, the data cleaning process, eliminate data outliers and acquisition error value. When the model is established, the data satisfying the normal distribution is more likely to reflect the characteristics and improve the fitting accuracy of the model. According to the requirement of normal distribution, the value of *y* is almost all concentrated in the (μ − 3σ, μ + 3σ) interval, that is, the 3σ criterion should be satisfied. Any error exceeding this interval is not a random error but a gross error, and the data containing the error should be eliminated. According to the Bessel formula, the σ and μ of the normal distribution are calculated.

12 $$\sigma = \left[ {\frac{1}{n - 1}\sum\limits_{i = 1}^{n} {v_{i}^{2} } } \right]^{1/2} = \left\{ {\left[ {\sum\limits_{i = 1}^{n} {x_{i}^{2} - \left( {\sum\limits_{i = 1}^{n} {x_{i} } } \right)^{2} /n} } \right]/(n - 1)} \right\}^{1/2}$$

The restrictive variables of each variable are calculated by Matlab, and the results are shown in Table 6:

**Table 6:** Values of σ calculated.

Variables	Sigma value	Variables	Sigma value
*f*-507	187.89	*f*-1528	117.15
*f*-520	306.93	*f*-1727	237.06
*f*-839	115.81	*f*-1826	336.92
*f*-1073	136.33	Temperature	57.97
*f*-1371	1094.4	C contents	39.20

**Figure 10:**
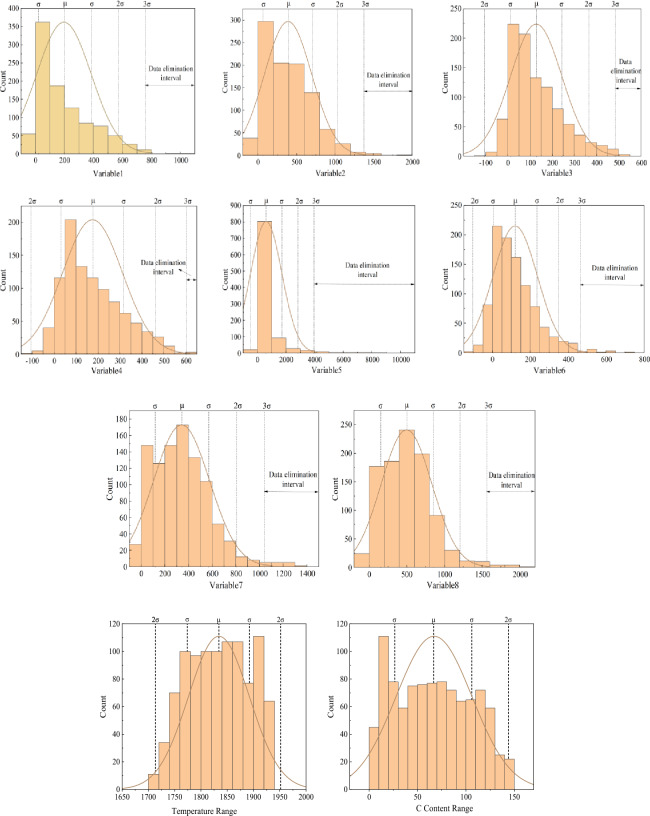
Elimination of data intervals for each indicator.

Through the (μ − 3σ, μ + 3σ) interval, the abnormal value data is eliminated. As shown in Fig. 10. It can be found that the record of independent variables exists outliers, need to eliminate outliers; as the dependent variables, temperature and carbon content tend to be more normal distribution, no obvious outliers.

### Model solution and verification

**Figure 11:**
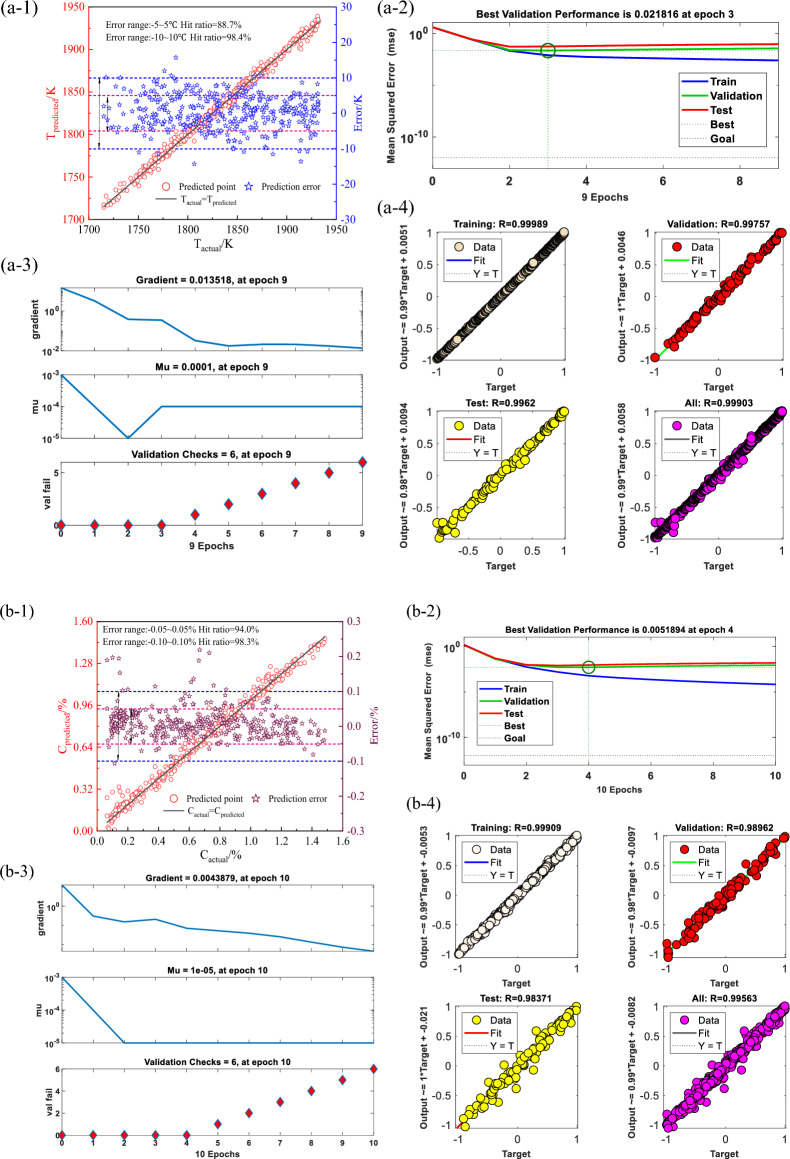
BP neural network model predicts temperature and carbon content results. (**a-1**) Temperature test set prediction results; (**a-2**) Neural network temperature prediction model accuracy evolution; (**a-3**) Neural network temperature prediction model iteration process; (**a-4**) Training set, test set, validation set, full set prediction accuracy; (**b-1**) Carbon content test set prediction results; (**b-2**) Neural network carbon content prediction model accuracy evolution; (**b-3**) Neural network carbon content prediction model iteration process; (**b-4**) Training set, test set, validation set, full set prediction accuracy.

According to the training principle of BP neural network, 2/3 data is selected for training and 1/3 data is selected as the test set, at the same time, the BP neural network algorithm model will independently select a part of the data in the training set as the verification set to verify the generalization ability of the model. To avoid overfitting caused by data order, the original 978 sets of data are disrupted, 600 sets of data are selected as the training set, and 378 sets of data are selected as the test set. The number of iterations of the neural network is set to 1000, the convergence error is set to 10^–12^, and the learning rate is set to 1. The results shown in Fig. 11 are obtained by calculation.

The BP neural network of temperature prediction model converges after 4 iterations, and the model converges at a convergence error of 0.0218. After 6 error verifications, it is found that the convergence error remains unchanged or even increases. Through the calculation of R^2^, it is found that the prediction accuracy of the training set is 0.99, the prediction accuracy of the test set is 0.99, the prediction accuracy of the verification set is 0.99, and the prediction accuracy of the whole set is 0.99. The model prediction accuracy is high, indicating that the application of BP model can well express the prediction relationship between each variable and temperature value, and can achieve the purpose of intelligent prediction. Through statistics, it is found that the hit rate of the temperature model in the range of ± 5 K is 88.7%, and the hit rate in the range of ± 10 K is 98.4%, and the *RMSE* parameter analysis shows that the average prediction error is 3.85 K which can fully meet the requirements of field prediction. The BP neural network of carbon content prediction model converges after 2 iterations, and the model converges at a convergence error of 0.0052. After 6 error verifications, it is found that the convergence error remains unchanged or even has an increasing trend. Therefore, it stops at 10 iterations and converges faster; through the calculation of R^2^, it is found that the prediction accuracy of the training set is 0.99, the prediction accuracy of the test set is 0.99, the prediction accuracy of the verification set is 0.98, and the prediction accuracy of the whole set is 0.99. The model prediction accuracy is high, indicating that the application of BP net model can well express the prediction relationship between each variable and carbon contents value, and can achieve the purpose of intelligent prediction. Through statistics, it is found that the hit rate of the carbon contents model in the range of ± 0.05% is 94%, and the hit rate in the range of ± 0.10% is 98.3%, and the *RMSE* parameter analysis shows that the average prediction error is 0.02% which can fully meet the requirements of field prediction.

### Model universality and accuracy analysis

In order to explain the impact of indicators more intuitively and verify the universality of the model, MIV analysis is used to quantify the impact of indicators. The specific method is as follows: for the trained neural network, the independent variables of the training data are increased by 10% or decreased by 10%, and two new training data are obtained. The data are used to predict the results of the two groups. Assuming B1 and B2, the difference between B1 and B2 is obtained, which is called IV. Then, take the average, which is the mean—IV, the MIV value. The variable range is expanded by 10% and reduced by 10% respectively, and the prediction results shown in Table 7 are obtained.

**Table 7:** Model prediction results.

Model	Increase the range of 10% variables		Source data set		10% reduction in variable range	
	R^2^	RMSE	R^2^	RMSE	R^2^	RMSE
Temperature prediction model	0.99	3.87	0.99	3.85	0.99	3.81
Carbon content prediction model	0.99	0.022	0.99	0.021	0.99	0.020

It can be seen from the results that the variable range increases by 10% and decreases by 10% have a slight effect on the accuracy of the model, but the overall prediction accuracy of the model is higher, indicating that the BP neural network model will not be affected with the change of sample number and parameter range, that is, the universality of the model is higher. Increasing the range of 10% variables, the R^2^ of the temperature prediction model and the carbon content prediction model have no changes and the prediction error of *RMSE* becomes larger, indicating that the larger range of parameters brings challenges to the prediction accuracy, which is also the direction that needs to be extended in the future work. Reducing the range of 10%, the prediction accuracy of the model increases, the R^2^ value have no changes, and the prediction error of *RMSE* becomes smaller, indicating that the internal convergence of the model is strong, and the recognition of the small range of parameters is strong. In general, the universality of the model is strong, the model is more practical.

Through the statistics of the accuracy of the existing spectral data to predict the converter temperature and carbon content, the results shown in Table 8 are obtained.

**Table 8:** Comparison of model accuracy.

Carbon content hit rate (%)			Temperature hit rate (%)			Double hit rate (%)		
Model 1^19^	Model 2^17^	Model in this paper(± 0.1%)	Model 1^19^	Model 2^17^	Model in this paper(± 10 K)	Model 1^13^	Model 2^18^	This paper (± 0.1% ∩ ± 10 K)
93.3	83	98.3	93.3	90	98.4	90	89	96.7

By comparing the previous research results, it is found that the model adopted in this paper has improved the prediction accuracy of real carbon content, temperature prediction accuracy and double hit rate compared with the previous research, which provides a new model and idea for the application of spectral technology in steelmaking.

## Conclusions

Through the collection and processing of flame spectrum data in the process of converter smelting, the real-time prediction of converter smelting temperature and C content is carried out by rough set attribute reduction algorithm optimized by genetic algorithm and BP neural network algorithm. The following conclusions can be drawn.

1. The spectral baseline is fitted and removed by using the BEADS algorithm, and then through the attribute reduction algorithm to reduce the dimension of 2048-dimensional spectral data, eight indicators that have a greater contribution to temperature and carbon content are selected, which are *f*-507, *f*-520, *f*-839, *f*-1073, *f*-1371, *f*-1528, *f*-1727 and *f*-1826, respectively. The MIC coefficients of the eight indicators with temperature and carbon content are calculated, and the MIC coefficients of the variables is small, and the selected indicators are representative. There was a significant correlation between temperature and C content. The lower the C content, the higher the temperature.
2. In BP neural network of temperature prediction model, it is found that the prediction accuracy of the training set is 0.99, the prediction accuracy of the test set is 0.99, the prediction accuracy of the verification set is 0.99, and the prediction accuracy of the whole set is 0.99. Through statistics, it is found that the hit rate of the temperature model in the range of ± 5 K is 88.7%, and the hit rate in the range of ± 10 K is 98.4%. and the *RMSE* parameter analysis shows that the average prediction error is 3.85 K.
3. In BP neural network of carbon content prediction model, it is found that the prediction accuracy of the training set is 0.99, the prediction accuracy of the test set is 0.99, the prediction accuracy of the verification set is 0.98, and the prediction accuracy of the whole set is 0.99. Through statistics, it is found that the hit rate of the carbon contents model in the range of ± 0.05% is 94.0%, and the hit rate in the range of ± 0.10% is 98.3%, and the *RMSE* parameter analysis shows that the average prediction error is 0.021%.
4. The universality of the model is verified by MIV algorithm. Increasing the range of 10% variables, and the prediction error of *RMSE* becomes larger, while reducing the range of 10%, the prediction accuracy of the model increases, and the prediction error of *RMSE* becomes smaller, so the universality of the model is strong, the model is more practical.

## Data Availability

Due to the nature of this research, participants of this study did not agree for their data to be shared publicly, so supporting data is not available. If you need data, you can contact the first author.
